# Impact of interaction between an artificial intelligence endoscopic support system and endoscopists on diagnosis of gastric neoplastic lesions

**DOI:** 10.1055/a-2695-0556

**Published:** 2025-10-09

**Authors:** Hiroya Mizutani, Yosuke Tsuji, Dai Kubota, Hiroyuki Hisada, Yuko Miura, Daisuke Ohki, Chihiro Takeuchi, Naomi Kakushima, Nobutake Yamamichi, Ryosuke Kikuchi, Mitsuaki Ishioka, Atsuo Yamada, Shinya Kodashima, Tomohiro Tada, Mitsuhiro Fujishiro

**Affiliations:** 1 13143 Department of Gastroenterology Graduate School of Medicine, The University of Tokyo, Bunkyo-ku, Japan; 2 13143 Department of Next-Generation Endoscopic Computer Vision Graduate School of Medicine, The University of Tokyo, Bunkyo-ku, Japan; 3 AI Medical Service Inc., Tokyo, Japan; 4 117105 Department of Gastroenterology, The Cancer Institute Hospital Of JFCR, Koto-ku, Japan; 5 Nihonbashi Ningyocho Gastroenterology and Endoscopy Clinic, Tokyo, Japan; 6 Ochanomizu Surugadai Clinic, Tokyo, Japan; 7 Department of Medicine Graduate School of Medicine, Teikyo University School of Medicine, Tokyo, Japan; 8 13143 Department of Surgical Oncology Graduate School of Medicine, The University of Tokyo, Bunkyo-ku, Japan; 9 Department of Gastroenterology and Proctology, Tada Tomohiro Institute of Gastroenterology and Proctology, Saitama, Japan

**Keywords:** Endoscopy Upper GI Tract, Precancerous conditions & cancerous lesions (displasia and cancer) stomach, Diagnosis and imaging (inc chromoendoscopy, NBI, iSCAN, FICE, CLE)

## Abstract

**Background and study aims:**

Artificial intelligence (AI) is expected to enhance the ability of endoscopists to detect gastric neoplastic lesions; however, its effectiveness among highly skilled Japanese expert endoscopists has not been validated. We developed a novel AI-assisted diagnostic tool for detection of gastric neoplastic lesions and evaluated its utility by comparing the diagnostic performance of endoscopists with and without AI assistance.

**Patients and methods:**

Diagnostic performance of gastric neoplastic lesions without and with AI assistance was compared among 14 expert endoscopists and 12 non-expert endoscopists using an evaluation dataset consisting of 150 images containing neoplastic lesions and 350 images without lesions. A general linear mixed model was applied for comparative analysis. The primary outcome was to demonstrate superiority of sensitivity and non-inferiority of specificity among expert endoscopists using AI compared with those without AI. The significance level for sensitivity was set at 2.5% and the non-inferiority margin for specificity was defined as a log odds ratio of –0.25.

**Results:**

Our AI demonstrated superiority in sensitivity (66.4% without AI vs. 83.5% with AI; odds ratio [OR] 2.562, 97.5% confidence interval [CI] 2.069–3.172) and non-inferiority in specificity (90.8% without AI vs. 92.9% with AI; OR 1.326, 95% CI 1.122–1.565) among expert endoscopists.

**Conclusions:**

AI contributed to improved diagnostic performance even among Japanese expert endoscopists in detecting gastric neoplastic lesions. These findings suggest that the AI system may have potential to support consistently high diagnostic performance across varying levels of endoscopic expertise.

## Introduction

Gastric cancer remains the third leading cause of world-wide cancer-related mortality, accounting for 768,000 deaths reported in 2020, and in particularly, it is the leading cause of death in several Asian countries 1 . Gastrointestinal endoscopy is regarded as the gold standard for early detection of gastric cancer and precancerous lesions. In Japan, it has been incorporated into a nationwide screening program since 2016. However, studies have reported that approximately 9.7% of gastric cancers are missed during upper gastrointestinal endoscopy 2 , with diagnostic accuracy highly dependent on endoscopist skill and experience.

Artificial intelligence (AI) based on convolutional neural networks (CNNs) for image recognition plays a substantial role in addressing variability in diagnostic accuracy arising from differences in endoscopic skill and experience. In gastric cancer detection, Hirasawa et al. (2018) developed a CNN diagnostic system using 13,584 gastric cancer images for training data and reported excellent diagnostic performance with a sensitivity of 92.2% 3 . Many studies since then have corroborated the high diagnostic accuracy of AI for gastric cancer detection 4 5 6 7 8 . However, benefits of AI in routine clinical practice remain inconclusive. Although AI diagnostic accuracy is undoubtedly important in clinical practice, it functions solely as an adjunct to the endoscopist, who is responsible for interpreting and selecting AI-generated diagnostic information in the decision-making process. An endoscopist’s response to AI diagnosis suggestions depends not only on AI performance but also on various factors such as AI interface design and usability and other complex factors, as well as the endoscopist’s competencies, experience, and psychological background. Therefore, in development and optimization of AI, the effect of endoscopist-AI interaction should be considered and evaluated. In this study, we aimed to simulate and assess the effect of endoscopist-AI interaction by comparing image-based diagnostic sensitivity and specificity for gastric neoplastic lesions between endoscopists using AI and those without AI on the same evaluation image dataset.

Few studies have validated whether AI enhances endoscopist ability to detect gastric cancer, with most studies utilizing magnifying endoscopy with image-enhanced endoscopy 9 10 11 12 13 . Although a few studies have performed validation using white light imaging (WLI) 14 15 , they were conducted in China and none in Japan. Based on these reports, the early detection rate of gastric cancer in China is approximately 5% to 20%, which is considerably lower compared with that in Japan (75%) 15 16 17 18 . Therefore, it is crucial to assess whether diagnostic capabilities of AI can provide additional benefits, even for experienced Japanese endoscopists with extensive expertise in early gastric cancer detection, providing important insights into evaluating the effectiveness of AI-assisted endoscopy.

## Patients and methods

### Model development based on CNN

An endoscopic AI system for diagnosing early gastric cancer and gastric epithelial neoplastic lesions, including adenoma under WLI, was constructed using You Only Look Once X (YOLOX), a real-time object detection algorithm 19 . We used 51,007 endoscopic images obtained from 50 medical institutions as the training data and 6,406 endoscopic images as the validation data. This AI model is designed to detect gastric neoplastic lesions by predicting bounding boxes and their confidence scores for identified objects on input images resized to 512 × 512. Non-maximum suppression 20 was applied to the predicted bounding boxes to eliminate duplicate detections and select the most relevant detected objects. Bounding boxes with confidence scores that exceeded the predefined threshold (0.3) were then extracted as AI diagnostic outputs and superimposed onto the endoscopic image. Non-neoplastic lesions such as gastritis, atrophic gastritis, polyps, and gastric ulcers, including scars, were excluded from detection. Training data details used for AI construction are presented in Table 1 .

**Table:** **Table 1** Findings included in training and evaluation datasets used for AI development.

	Training data	Validation data
Early gastric cancer		
Type 0-I (protruding)	995	70
Type 0-iia (superficial elevated)	9008	907
Type 0-iib (superficial flat)	1601	171
Type 0-iic (superficial depressed)	18450	2111
Type 0-III (depressed)	45	0
Advanced gastric cancer		
Type 1	280	0
Type 2	1079	145
Type 3	1380	281
Type 4	186	29
Type 5	24	0
Gastric adenoma	1845	150
Gastric ulcer	2009	187
Gastric ulcer scar	1486	232
Erosion	4761	542
Hyperplastic polyp	2282	289
Fundic gland polyp	3	0
Gastritis	781	93
Background gastric mucosa	4792	1199
Total	51007	6406
AI, artificial intelligence.		

### Study design

This was an open-label, comparative validation study. The endoscopic images for the evaluation dataset used in this study were generated from upper gastrointestinal endoscopy videos collected from six institutions in studies registered in the Japan Registry of Clinical Trials and written informed consent was provided for use of the data. This research was approved by the Institutional Review Board of University of Tokyo Hospital.

### Evaluation dataset

Three endoscopists with ≥ 10 years of experience in upper gastrointestinal endoscopy and with board certification were designated as "gold-standard endoscopists" to create the evaluation dataset for the diagnostic performance test. These endoscopists assigned diagnostic information regarding gastric neoplastic lesions to the endoscopic images following a defined procedure. Only images and diagnostic information on which all three gold-standard endoscopists agreed were included in the evaluation dataset, along with their consensus-based gold-standard answers. Detailed selection requirements for gold-standard endoscopists are presented in Table 2 .

**Table:** **Table 2** Selection criteria for gold-standard, expert, and non-expert endoscopists.

	Gold-standard endoscopist	Expert endoscopist	Non-expert endoscopist
Board certification by the Japan Gastroenterological Endoscopy Society	Board-certified trainer	Board-certified fellow	Not certified
Experience with upper gastrointestinal endoscopy	≥ 10 years	≥ 5 years	6 months-5 years
Average annual number of upper gastrointestinal endoscopies performed in the last 5 year	≥ 200	≥ 200	Unregulated
Number of upper gastrointestinal endoscopies in the last 1 year	≥ 200	≥ 200	Unregulated
Physician specialty	Gastroenterology	Gastroenterology	Unregulated

From the upper gastrointestinal endoscopic videos collected from six medical institutions, 21,281 still images were extracted, of which 14,319 were obtained by excluding non-stomach images. All images and videos were obtained using Olympus endoscopes (GIF-H290Z, GIF-H290, GIF-HQ290, and GIF-XZ1200; Olympus Medical Systems, Tokyo, Japan) and video processors (EVIS LUCERA ELITE CV-290 and EVIS X1 CV-1500; Olympus Medical Systems). These images were organized into three distinct image sets, each assigned to one of three gold-standard endoscopists. Each gold-standard endoscopist reviewed their designated image set with reference to the original videos and applied a bounding box and diagnostic information for any detected gastric neoplastic lesions. For images without identified gastric neoplastic lesions, only diagnostic information was added. During this process, images taken under conditions other than non-magnified WLI were excluded. Images that did not capture the entire target lesion, contained redundant views of previously identified lesions, and those taken under suboptimal conditions (such as contamination by mucus or blood, out-of-focus images, blurs, bubbles, halation, or inappropriate angles/distances) were excluded. Moreover, images containing suspected non-epithelial lesions or advanced cancers were excluded. The bounding box and diagnosis data provided by each gold-standard endoscopist were independently verified by two other gold-standard endoscopists. Images lacking unanimous agreement between the three gold-standard endoscopists (54 images) were excluded.

**Fig. 1:**
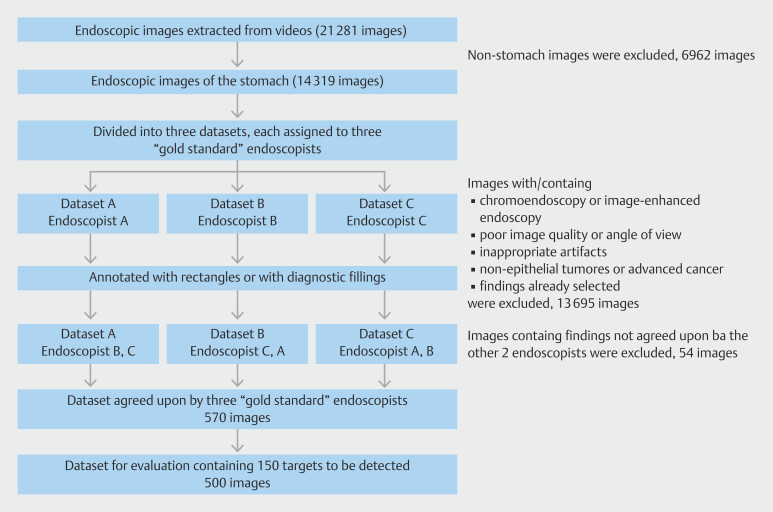
Flowchart for creating evaluation dataset. Based on the consensus of three gold-standard endoscopists, a total of 500 images were selected as the evaluation dataset: 150 images containing the detection target and 350 images without it.

Of the 570 images registered as described above, 150 images with the target lesion and 350 images without the target lesion were selected to create the evaluation dataset, prioritizing images from the most recent examination dates. In this selection process, to ensure generalizability across various findings and examination conditions, the final dataset was arranged to include at least 10 images for each of the following findings: early gastric cancer (elevated, flat, and depressed types), adenomas, atrophic gastritis or gastritis, polyps, and gastric ulcers or scars. In addition, at least 30 images from each endoscope model (Olympus 290 series and 1200 series) and video processor (EVIS LUCERA ELITE CV-290 and EVIS X1 CV-1500) were selected. Ultimately, 500 images—150 with the detection target and 350 without—were finalized as the evaluation dataset. The process for creating the evaluation dataset is illustrated in Fig. 1 .

### Diagnostic test

**Fig. 2:**
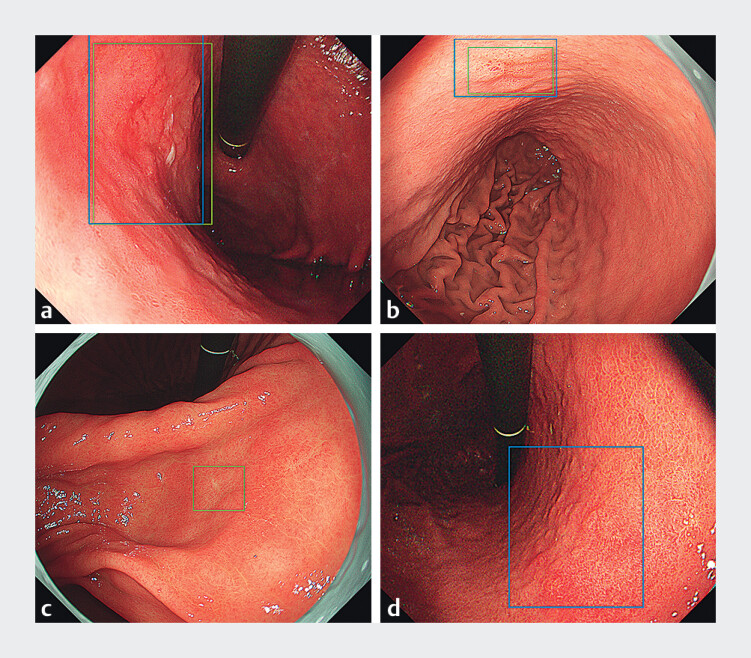
Representative images of AI reading results in diagnostic tests. Yellow bounding boxes indicate areas of gastric neoplastic lesions annotated as gold-standard answers. Blue bounding boxes indicate areas where the AI diagnosed as gastric neoplastic lesions. **a,b** Examples of correct answers by AI. The bounding boxes identified by AI closely aligned with the gold-standard annotations, achieving a Dice coefficient exceeding 0.3. **c** False-negative case by AI. AI did not detect a lesion indicated by gold-standard annotation. **d** False-positive case by AI. AI recognized atrophic gastric mucosa as a neoplastic lesion.

First, to evaluate performance of the AI system, diagnostic results were generated for all images in the evaluation dataset. For each image, AI estimated presence or absence of the detection target. If present, it indicated its range with bounding boxes on the images. AI diagnoses were compared with the gold-standard answers and judged as correct or incorrect. For the detection target, the bounding box estimated by AI was compared with the gold-standard bounding box and judged as correct when the Dice coefficient 21 was ≥ 0.3, and incorrect when it was < 0.3. The Dice coefficient is defined as 2C/(A+B), where A is the area of the gold-standard bounding box, B is the area of the bounding box estimated by AI, and C is the area of overlap between the two bounding boxes. Representative images of AI reading results in diagnostic tests are presented in Fig. 2 .

Subsequently, 14 expert endoscopists (board-certified fellows with ≥ 5 years of experience performing at least 200 upper gastrointestinal endoscopies per year) and 12 non-expert endoscopists (non-certified fellows with 6 months to < 5 years of upper gastrointestinal endoscopy experience) were prepared to evaluate the effect of using AI. The endoscopists first reviewed all images in the evaluation dataset to estimate presence or absence of the target lesion and annotated the detected lesions with bounding boxes without AI assistance (without AI). They then repeated the same test for the same dataset with reference to diagnoses provided by AI (with AI). Endoscopist results were compared with the gold standard and judged correct or incorrect. Dice coefficients ≥ 0.3 were considered correct and those <0.3 were deemed incorrect for the bounding boxes. Detailed selection criteria for the expert and non-expert endoscopists are presented in Table 2 .

### Outcome

The primary outcome was comparison of sensitivity and specificity between “without AI” and “with AI” in the expert endoscopist group. The secondary outcome included a comparison of sensitivity and specificity between “without AI” and “with AI” in all endoscopists, “without AI” and “with AI” in the non-expert endoscopist group, and AI alone and expert endoscopists without AI.

### Statistical analysis

For the primary outcome, which compared sensitivity and specificity between “without AI” and “with AI” in the expert endoscopist group, a general linear mixed model (GLMM) was applied to the data subset where the gold-standard was positive, that is, detection targets were included, and the endoscopist category was “expert endoscopists.” The log odds ratio for sensitivity of “with AI” over “without AI” was calculated, along with the corresponding odds ratio (OR) and its two-sided 97.5% confidence interval (CI). The two-sided *P* value (significance level 2.5%) corresponding to the null hypothesis “H0: odds ratio = 1” was also calculated to verify superiority of sensitivity of “with AI” over “without AI.” Sensitivity in “without AI” was calculated based on simple frequencies, and that of “with AI” was estimated using the OR and the sensitivity of “without AI.”

To compare specificity between “without AI” and “with AI” in the expert endoscopist group, GLMM was applied to the data subset where the gold standard was negative, that is, no detection targets were present, and the endoscopist category was “expert endoscopists.” The log OR for specificity of “with AI” over “without AI” was calculated and the corresponding OR, along with its two-sided 95% CI, were estimated. The non-inferiority margin was set at a log OR of -0.25. To test the null hypothesis, H0: OR ≤ exp (-0.25) ≈ 0.7788, we calculated a one-sided *P* value at a 2.5% significance level to verify the non-inferiority of specificity of “with AI” over “without AI.” The specificity of “without AI” was calculated based on simple frequencies and the sensitivity of “with AI” was estimated based on the OR and specificity of “without AI.”

For secondary outcomes, similar to the primary outcome, GLMM was applied to calculate the log OR for sensitivity/specificity of “with AI” over “without AI” in all and the non-expert endoscopist group, and for sensitivity/specificity of “expert endoscopists without AI” over “AI alone.” ORs and their CIs were calculated. Sensitivity/specificity of “without AI” was calculated based on simple frequencies, whereas that of “with AI” was estimated based on the OR and sensitivity/specificity of “without AI.” Similarly, the sensitivity/specificity of “AI alone” was calculated based on simple frequencies, whereas that of “expert endoscopists without AI” was estimated based on OR and sensitivity/specificity of “AI alone.”

In this study, given that multiple endoscopists assessed the same image set, GLMM was applied to analyze sensitivity and specificity using ORs. This method was selected to enhance robustness and reliability of the results while mitigating potential effects of interobserver correlation among the endoscopists. To determine the non-inferiority margin, risks and benefits of AI use were carefully considered. The primary risk associated with AI use is potential influence of false-positive results on endoscopists, leading to an increase in unnecessary biopsies. Conversely, the benefit of AI lies in its ability to enhance endoscopist sensitivity, thereby reducing the likelihood of missing gastric epithelial neoplastic lesions. Taking these benefits into consideration, deterioration in specificity of expert endoscopists of up to 3.0% due to AI use was deemed acceptable. Based on findings from our previous pilot study, the expected specificity of expert endoscopists (without AI) was estimated to be 87.3%, with an anticipated specificity difference of approximately 3% before and after AI implementation when the log OR of specificity was -0.25. Therefore, the non-inferiority margin was set with a log OR of -0.25.

SAS Software v. 9.4 (SAS Institute, Cary, North Carolina, United States) and R Statistical Software v. 4.2.2 (R Core Team 2022) were used for statistical analysis.

## Results

### Primary outcome

Sensitivity of “without AI” in the expert endoscopist group, calculated based on simple frequencies, was 66.4%. Conversely, sensitivity of “with AI”, estimated using OR, was 83.5% (97.5% CI: 80.35-86.24). Difference in sensitivity between “with AI” and “without AI” was +17.1% (97.5% CI 13.95–19.84), indicating a significant increase in sensitivity when using AI among expert endoscopists.

Furthermore, although specificity of “without AI” in the expert endoscopist group, calculated based on simple frequencies, was 90.8%, specificity of “with AI”, estimated using OR, was 92.9% (95% CI 91.70–93.91). The difference in specificity between “with AI” and “without AI” was +2.1% (95% CI 0.93–3.13). The *P* value for OR ≤ exp (-0.25) was < 0.001, indicating non-inferiority of specificity with AI use among expert endoscopists ( Table 3 ).

**Table:** **Table 3** Comparison of diagnostic performance without AI and with AI.

		Without AI	With AI	Odds ratio	Confidence interval (CI)	*P* value
**Expert endoscopists**						
Sensitivity		66.4	83.5	2.562	97.5% CI 2.0695–3.1721	< 0.001
Specificity		90.8	92.9	1.326	95% CI 1.1228–1.5657	< 0.001
**All endoscopists**						
Sensitivity		63.8	81.7	2.536	95% CI 2.2180–2.8997	–
Specificity		90.1	91.5	1,186	90% CI 1.0699–1.3149	–
**Non-expert endoscopists**						
Sensitivity		60.7	80.3	2.638	95% CI 2.1720–3.2041	–
Specificity		89.3	89.8	1.054	90% CI 0.9082–1.2222	–
		**AI alone**	**Expert without AI**	**Odds ratio**	**Confidence interval (CI)**	
**AI alone vs. expert**	Sensitivity	91.4	43.7	0.073	90% CI: (0.0404–0.1303)	–
	Specificity	90.3	90.9	1.076	90% CI: (0.7587–1.5266)	–
AI, artificial intelligence.						

### Secondary outcome

Sensitivity of “with AI” estimated using OR was 81.7% (95.0% CI 79.61–83.61) among all endoscopists, compared with 63.8% for “without AI” based on simple frequencies. This indicates superior sensitivity with AI use among all endoscopists. Specificity of “with AI”, estimated using OR, was 91.5% (90% CI 90.69–92.29) among all endoscopists, compared with 90.1% for “without AI” based on simple frequencies. This suggests non-inferiority of specificity with AI use among all endoscopists.

Sensitivity of “with AI”, estimated using OR, was 80.3% (95.0% CI 77.03–83.18) among non-expert endoscopists, compared with 60.7% for “without AI” based on simple frequencies, indicating superiority of sensitivity with AI use among non-expert endoscopists. Specificity of 89.8% (90% CI 88.35–91.08) for “with AI”, estimated using OR, was almost equivalent to 89.3% for “without AI” based on simple frequencies among non-expert endoscopists.

Sensitivity of “expert endoscopists without AI”, estimated using OR, was 43.7% (90.0% CI 30.19–58.22), compared with 91.4% for “AI alone” based on simple frequencies, indicating superior sensitivity of “AI alone” over “expert endoscopists without AI”. Specificity of 90.9% (90% CI 87.58–93.42) for “expert endoscopists without AI”, estimated using OR, was almost equivalent to 90.3% for “AI alone” based on simple frequencies ( Table 3 ).

## Discussion

In this study, we examined the impact of our endoscopic AI system on endoscopist accuracy in diagnosing gastric neoplastic lesions, including early gastric cancer and gastric adenoma, using an evaluation dataset consisting of 500 endoscopic images created by three gold-standard endoscopists. This examination was performed on 14 expert endoscopists and 12 non-expert endoscopists. Our findings showed that sensitivity of “with AI” was significantly higher than that of “without AI” in all endoscopist categories, indicating that AI diagnostic assistance improves detection of gastric neoplastic lesions by both expert and non-expert endoscopists. Furthermore, the results demonstrated that specificity of “with AI” was non-inferior to, and even surpassed, that of “without AI” among expert endoscopists. In addition, specificity of “with AI” exceeded that of “without AI” in all endoscopists and the non-expert endoscopist group, suggesting non-inferiority of specificity of “with AI”.

Zhang et al. 14 reported that in a study of endoscopic image-based examination under non-magnified WLI conditions, AI-assisted endoscopy demonstrated improved specificity in diagnosis of gastric neoplastic lesions among endoscopists with intermediate experience; however, no additive effect was observed in sensitivity among expert endoscopists. Selection of relatively difficult-to-diagnose gastric neoplastic lesions in their study was mentioned as a potential reason for lack of efficacy in the expert group. However, sensitivity and specificity of the expert group without AI in their study (67.8% and 85.8%, respectively) were largely equivalent to those in our study (66.4% and 90.8%, respectively). In our study, we confirmed a significant improvement in both sensitivity and specificity among experts with AI support, indicating that our AI system has greater potential to enhance diagnostic performance. Tang et al. 15 demonstrated that AI use significantly improved diagnostic performance in both expert and non-expert endoscopists, reporting extremely high sensitivity and specificity of 93.0% and 97.7%, respectively, for AI alone. However, given that the experts already exhibited favorable diagnostic accuracy (82.7% and 91.9%, respectively) without AI assistance, a direct comparison may not be appropriate, owing to variations in diagnostic complexity of the selected cases.

The European Society of Gastrointestinal Endoscopy position statement explicitly emphasizes that the expected role of AI is to instantly elevate the level of performance of less experienced endoscopists and improve the detection rate of gastrointestinal neoplasia dramatically and universally. In this study, AI successfully improved sensitivity in the non-expert endoscopist group and, therefore, is expected to effectively reduce the rate of missed early gastric neoplastic lesions. In addition, although low specificity is often cited as a challenge for AI endoscopy, specificity of AI alone in this study was sufficiently high at 90.3%, showing non-inferiority of specificity with AI in the expert endoscopist group. This is also expected to reduce excessive histological examination associated with unnecessary biopsies during endoscopy.

In this study, we validated the impact of AI assistance in endoscopic diagnosis of gastric neoplastic lesions under WLI conditions, considering the interaction between the endoscopist and AI and demonstrating its efficacy irrespective of endoscopist experience level. To our knowledge, no similar study has been conducted in Japan. The fact that AI assistance demonstrated an additional benefit even among highly skilled Japanese expert endoscopists well-versed in gastric cancer diagnosis suggests that AI-assisted endoscopy has the potential to mitigate geographical and technical disparities in endoscopic practice. It also provides universal access to advanced endoscopic diagnostic capabilities.

This study had some limitations. First, images in the validation dataset are subject to selection bias. During dataset preparation, suboptimal images were excluded, resulting in images in which lesions were captured under ideal conditions. Second, prevalence of gastric neoplastic lesions in this study differs markedly from that in real clinical practice, where such lesions are less prevalent than in the evaluation dataset. Nevertheless, as noted, specificity of this AI was sufficiently high, and specificity among expert endoscopists with AI assistance was non-inferior to and significantly better than that without AI. Therefore, we believe this discrepancy does not substantially impact the positive diagnosis rate.

Another important limitation of this study is that histopathological confirmation was not used in establishing the reference standard of evaluation dataset. This may leave room for potential diagnostic inaccuracy in the ground truth. However, to minimize subjectivity and enhance reliability of the reference standard, we employed a consensus-based approach among three expert endoscopists (gold-standard endoscopists). Only findings agreed upon by all three were adopted, thereby ensuring a high level of objectivity and internal validity. Moreover, the primary aim of the AI model developed in this study was to detect endoscopic findings that suggest neoplastic lesions or indicate need for biopsy, rather than to make definitive histopathological diagnoses. Therefore, we believe that absence of pathological confirmation does not substantially compromise validity or clinical relevance of the study design.

In addition, although we simulated human-AI interaction by comparing diagnostic performance of endoscopists with and without the assistance of the AI system, actual human-AI interaction in clinical practice is subject to far more complex and variable influences. Real-world utility of AI is not determined solely by its algorithmic performance; rather, it is shaped by a range of factors, including the user interface design, clinical environment, task complexity, and cognitive biases of the endoscopist.

Indeed, discrepancies between the AI and endoscopists were frequently observed in this study. Specifically, there were 1,258 cases in which the AI suggested a positive finding, but the endoscopist judged it as negative—accounting for 28.0% of all cases that endoscopists diagnosed as negative. Conversely, in 514 cases (6.0% of all endoscopist-positive diagnoses), the AI output was negative whereas the endoscopist judged the finding as positive. These discrepancies highlight the complexity of human-AI collaboration and suggest that decision-making is influenced not only by AI outputs but also by human interpretation and confidence levels.

Such divergences underscore the difficulty of comprehensively simulating or evaluating human-AI interaction through image-based experimental settings alone. To better understand the practical impact of AI systems, further studies conducted in real-time clinical environments are needed, and we are planning a multicenter prospective study to address these limitations.

Finally, all endoscopic images in the evaluation dataset for this study were obtained using endoscope and video processor systems manufactured by Olympus; thus, their diagnostic performance relative to those acquired with Fujifilm or other endoscopic systems remains unknown.

## Conclusions

In conclusion, our AI system significantly enhanced diagnostic accuracy of gastric neoplastic lesions, demonstrating improvements in both sensitivity and specificity, even among expert endoscopists in Japan. These results substantiate the promising performance and potential effectiveness of the AI-based diagnostic support system in clinical practice.
